# Supplementary material for: Colorectal Cancer Anatomical Site and Sleep Quality
Source: Cancers (Basel). 2021 May 25;13(11):2578. doi: 10.3390/cancers13112578 (PMC8197388; doi:10.3390/cancers13112578)
Supplement: Supplementary file 1 [file cancers-13-02578-s001.zip › cancers-1201081-supplementary.pdf]

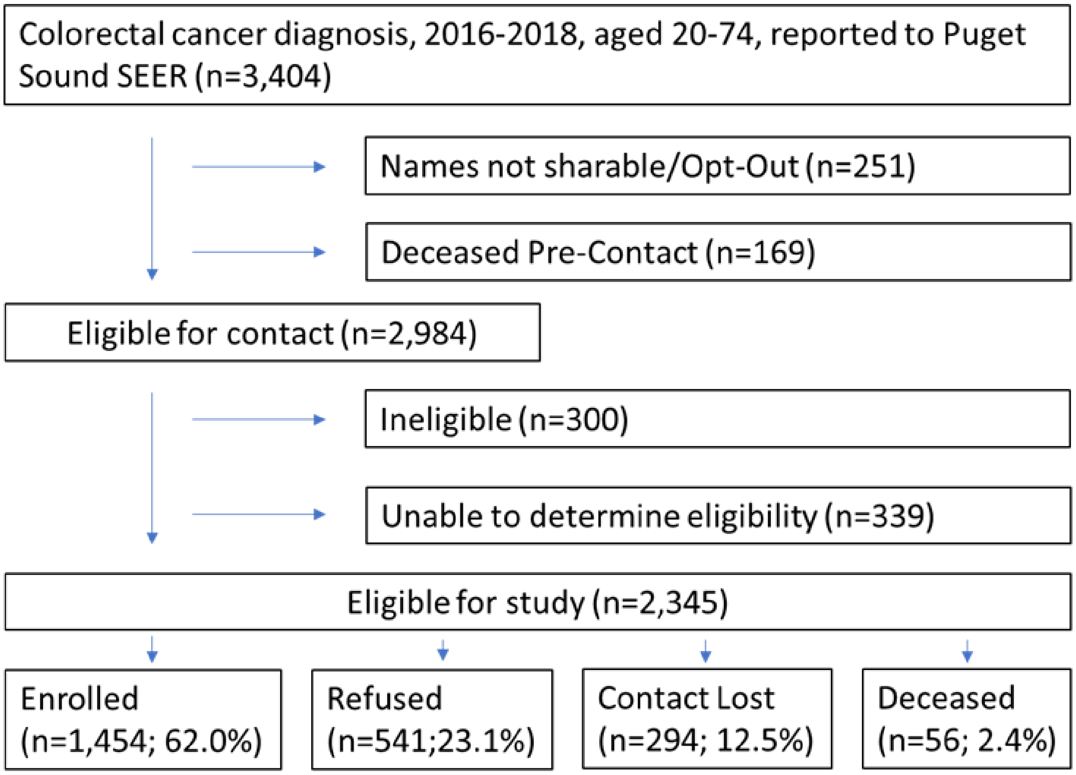

Supplemental Figure S1. CONSORT diagram for ACCESS study recruitment.

Supplemental Table S1. Association of sleep quality among regional and distant stage cancer patients compared to localized stage cancer patients (N=1453).

		Unadjusted OR (95% CI)	Adjusted* OR (95% CI)
Sleep Duration			
<7 hours			
	Regional	0.89 (0.70, 1.13)	0.82 (0.64, 1.04)
	Distant	0.95 (0.70, 1.30)	0.85 (0.62, 1.16)
7-8 hours			
	Regional	0.75 (0.59, 0.94)	0.78 (0.62, 0.99)
	Distant	0.69 (0.52, 0.94)	0.75 (0.55, 1.01)
≥9 hours			
	Regional	2.17 (1.55, 3.02)	2.27 (1.62, 3.18)
	Distant	2.20 (1.47, 3.29)	2.32 (1.54, 3.51)
Problems Staying Awake			
	Regional	1.52 (1.08, 2.15)	1.43 (1.00, 2.02)
	Distant	1.46 (0.95, 2.26)	1.28 (0.82, 1.99)
Poor Sleep Quality			
	Regional	1.28 (0.96, 1.69)	1.19 (0.89, 1.58)
	Distant	1.40 (0.99, 1.98)	1.25 (0.87, 1.78)
Sleep Medication Use			
	Regional	1.53 (1.09, 2.16)	1.52 (1.08, 2.16)
	Distant	2.17 (1.43, 3.29)	2.13 (1.38, 3.27)
Trouble Sleeping			
	Any problems		
	Regional	1.25 (0.95, 1.64)	1.15 (0.87, 1.52)
	Distant	1.51 (1.05, 2.18)	1.38 (0.95, 2.01)
	No sleep within 30 minutes Regional		
	Distant	1.23 (0.89, 1.68)	1.13 (0.82, 1.56)
		2.08 (1.45, 2.99)	1.81 (1.25, 2.62)
	Waking up in middle of sleep		
	Regional	0.95 (0.73, 1.24)	0.89 (0.68, 1.17)
	Distant	1.01 (0.72, 1.41)	0.92 (0.65, 1.29)
	Use the bathroom		
	Regional	1.06 (0.82, 1.36)	1.05 (0.81, 1.35)
	Distant	0.81 (0.59, 1.13)	0.85 (0.61, 1.19)
	Pain		
	Regional	0.98 (0.70, 1.36)	0.92 (0.65, 1.28)
	Distant	1.09 (0.73, 1.64)	1.02 (0.67, 1.55)
	Breathing/Cough/Snore		
	Regional	0.80 (0.44, 1.44)	0.75 (0.41, 1.37)
	Distant	0.66 (0.29, 1.50)	0.55 (0.24, 1.27)
Change in Sleep			
	Regional	2.24 (1.66, 3.04)	2.15 (1.58, 2.94)
	Distant	2.65 (1.85, 3.80)	2.50 (1.73, 3.62)

*Adjusted for age at diagnosis (years), sex (male, female), education (high school or less, some college, college graduate or higher), cancer site (colon, rectal) and time since diagnosis (months).

Supplemental Table S2. Association of sleep quality among rectal cancer patients compared to colon cancer patients stratified by cancer stage (N=1453).

		**Unadjusted OR (95% CI)**	**Adjusted* OR (95% CI)**
Sleep Duration			
<7 hours			
	Localized	1.56 (1.07, 2.28)	1.47 (0.99, 2.16)
	Regional	1.13 (0.81, 1.59)	0.96 (0.67, 1.37)
	Distant	1.03 (0.60, 1.76)	1.01 (0.58, 1.75)
7-8 hours			
	Localized	0.69 (0.48, 1.00)	0.71 (0.48, 1.03)
	Regional	0.88 (0.64, 1.22)	1.02 (0.73, 1.43)
	Distant	0.96 (0.57, 1.61)	0.92 (0.54, 1.57)
≥9 hours			
	Localized	0.87 (0.48, 1.58)	1.00 (0.54, 1.88)
	Regional	1.03 (0.69, 1.52)	1.03 (0.69, 1.56)
	Distant	1.02 (0.55, 1.92)	1.15 (0.60, 2.22)
Problems Staying Awake			
	Localized	0.90 (0.49, 1.64)	0.91 (0.49, 1.67)
	Regional	1.03 (0.66, 1.59)	0.88 (0.56, 1.40)
	Distant	0.61 (0.28, 1.32)	0.53 (0.24, 1.17)
Poor Sleep Quality			
	Localized	1.88 (1.20, 2.94)	1.75 (1.10, 2.79)
	Regional	1.12 (0.77, 1.63)	0.97 (0.65, 1.42)
	Distant	1.16 (0.65, 2.08)	1.15 (0.63, 2.10)
Sleep Medication Use			
	Localized	1.24 (0.70, 2.18)	1.40 (0.77, 2.53)
	Regional	0.86 (0.55, 1.36)	0.93 (0.57, 1.52)
	Distant	1.32 (0.67, 2.59)	1.27 (0.63, 2.55)
Trouble Sleeping			
	Any problems		
	Localized	1.92 (1.22, 3.04)	1.87 (1.17, 3.00)
	Regional	1.37 (0.92, 2.05)	1.33 (0.88, 2.01)
	Distant	1.83 (0.90, 3.72)	1.53 (0.74, 3.19)
	No sleep within 30 minutes Localized		
	Regional	1.37 (0.82, 2.28)	1.26 (0.75, 2.13)
	Distant	1.17 (0.77, 1.79)	1.11 (0.72, 1.71)
		1.38 (0.78, 2.44)	1.15 (0.62, 2.10)
	Waking up in middle of sleep		
	Localized	1.38 (0.91, 2.10)	1.38 (0.89, 2.12)
	Regional	0.94 (0.65, 1.36)	0.86 (0.59, 1.27)
	Distant	1.53 (0.87, 2.69)	1.28 (0.70, 2.32)
	Use the bathroom		
	Localized	1.54 (1.03, 2.31)	1.66 (1.09, 2.51)
	Regional	1.25 (0.88, 1.77)	1.26 (0.88, 1.81)
	Distant	1.87 (1.06, 3.28)	2.10 (1.16, 3.81)
	Pain		
	Localized	1.46 (0.87, 2.46)	1.49 (0.87, 2.55)
	Regional	1.73 (1.09, 2.72)	1.58 (0.98, 2.54)
	Distant	1.57 (0.80, 3.08)	1.36 (0.67, 2.77)
	Breathing/Cough/Snore		
	Localized	0.77 (0.29, 2.00)	0.73 (0.28, 1.96)
	Regional	0.53 (0.21, 1.38)	0.39 (0.14, 1.08)
	Distant	0.64 (0.13, 3.22)	0.46 (0.09, 2.47)

Change in Sleep			
	Localized	2.12 (1.27, 3.55)	1.91 (1.12, 3.23)
	Regional	1.33 (0.93, 1.92)	1.23 (0.85, 1.80)
	Distant	1.23 (0.71, 2.13)	1.29 (0.73, 2.30)

*Adjusted for age at diagnosis (years), sex (male, female), education (high school or less, some college, college graduate or higher) and time since diagnosis (months).

Supplemental Table S3. Association of sleep quality among rectal cancer patients compared to colon cancer patients with chemotherapy and radiation data (N=1148).

	Unadjusted OR (95% CI)	Adjusted 2[a] OR (95% CI)
**Sleep Duration**		
<7 hours	1.39 (1.07, 1.81)	1.26 (0.90, 1.77)
7-8 hours	0.81 (0.63, 1.05)	0.92 (0.66, 1.27)
≥9 hours	0.85 (0.60, 1.20)	0.79 (0.50, 1.24)
**Problems Staying Awake**	0.98 (0.67, 1.41)	0.82 (0.51, 1.34)
**Poor Sleep Quality**	1.61 (1.20, 2.17)	1.32 (0.89, 1.94)
**Sleep Medication Use**	1.10 (0.76, 1.59)	1.31 (0.81, 2.10)
**Trouble Sleeping**		
Any problems	1.98 (1.42, 2.77)	1.80 (1.18, 2.73)
No sleep within 30 minutes	1.32 (0.96, 1.82)	1.10 (0.72, 1.66)
Waking up in middle of sleep	1.40 (1.06, 1.86)	1.40 (0.98, 2.01)
Use the bathroom	1.82 (1.39, 2.39)	1.85 (1.31, 2.63)
Pain	1.74 (1.24, 2.44)	1.55 (0.99, 2.41)
Breathing/Cough/Snore	0.58 (0.28, 1.23)	0.66 (0.27, 1.63)
**Change in Sleep**	1.50 (1.11, 2.03)	1.18 (0.79, 1.77)

[a] Adjusted for age at diagnosis (years), sex (male, female), BMI (kg/m^2), education (high school or less, some college, college graduate or higher), cancer stage at diagnosis (localized, regional, distant), chemotherapy (yes, no), radiation (yes, no) and time since diagnosis (months).
